# How intent to interact can affect action scaling of distance: reply to Wilson

**DOI:** 10.3389/fpsyg.2014.00513

**Published:** 2014-06-05

**Authors:** Tamer M. Soliman, Arthur M. Glenberg

**Affiliations:** Department of Psychology, Arizona State University, Tempe, AZ, USA

**Keywords:** culture, self-construal, embodied cognition, distance perception, motor effort

Soliman et al. (2013) set out to demonstrate how the bodily level of analysis can unify explanations in psychology. Our argument was that common sensorimotor mechanisms underlie many of the behavioral phenomena that are currently segregated as cognitive, social, or cultural. Toward that end, we re-characterized a cultural construct—self-construal along the dimension of independence and interdependence (Markus and Kitayama, 1991)—as reflecting degree of interaction with ethnically diverse others.

According to our cultural motor-effort hypothesis, the interdependence-independence continuum is in part determined by tuning sensorimotor behavior through interactions. Interdependents tune vocal, gestural, expressive facial patterns, as well as interactions in greeting, eating, walking, dancing, praying (and so on) with members of their in-groups. In contrast, independents tune their interactions with a more ethnically-diverse set of people. Consequently, interdependents, more so than independents, would anticipate greater motor effort when interacting with out-groups (vs. in-groups) because of poor tuning. Furthermore, reasoning from Proffitt and Linkenauger (2013) as well as Schnall et al. (2008), anticipated motor effort should lead to increased distance judgments. Thus we predicted, and found, that interdependents judge distance to in-group members as shorter than do independents.

Wilson (2013) questioned our application of Proffitt and Linkenauger and Schnall et al. As he notes, Proffitt's data (although not data from Schnall et al.) suggest that effects of anticipated motor effort are restricted to particular motor systems. Hence, Wilson reasoned, the anticipated effort in interacting should not affect scaling of distance when planning to walk. Here we address Wilson's reasoning by (a) pointing to several research projects that suggest leakage across motor systems rather than modularity, and (b) suggesting why previous data, importantly, Witt et al. (2004, 2010) did not observe this leakage.

As one example of leakage, consider data reported by Gentilucci et al. (2001). When reaching for a block, the larger the block, the wider people unintentionally open their mouths. In addition, the larger the block, the louder they pronounce syllables printed on the block.

Now consider in more detail retroactive motor contagion (RMC): the ubiquitous finding that if two action patterns are conjoined, planning of the second action influences planning of the first action. Demonstrations of RMC can be found in Adam et al. (2000), Khan et al. (2007, 2010), and Lajoie and Franks (1997).

The “end-comfort effect” can also be seen as a type of RMC. For example, the kinematics of the transport-to-grasp movement toward a bottle systematically vary depending on whether the bottle is later to be displaced to a different spot, is used to pour water into a glass, or is to be thrown away (Ansuini et al., 2008). Importantly, RMC can cross anatomical and neuro-representational boundaries within the motor system and shows coordination across different effectors. van der Wel and Rosenbaum (2007), for example, asked participants to locomote to a table, grasp a bottle, and then move it to another spot that was either close to or far from its initial location. The initial motor pattern (i.e., locomotion) was found to be influenced by the distal motor pattern (i.e., object transport). Namely, a participant's final step was on the side opposite to the direction of the forthcoming transport movement when that transport required one more step after grasping (see also Cockell et al., 1995 and Studenka et al., 2012).

Thus, modularity of the motor system at the anatomical and brain-representational level does not always hold at the functional level. Instead, conjoining two action patterns induces an informational flow across effectors and planned goals. Importantly, this influence holds whether one or different motor systems are involved in the sequence, and whether the goals planned are homologous (e.g., tapping followed by tapping) or different (i.e., locomoting then grasping). In short, these findings support our assumption that anticipated effort of interaction can affect anticipated effort to walk, and thereby affect distance judgments.

With the above as a backdrop, why then do Proffitt's data (e.g., Witt et al., 2004, 2010) seem to suggest modularity? One possibility is based on a subtle difference between the design of our experiments and the Witt et al. experiments. In Witt's experiments, the manipulation phase targets one motor system and then tests the effect of the manipulation on perceived distance as the participant intends to perform another task. For example, adapting Proffitt and Linkenauger's (2013) terminology, participants are adapted while temporarily turned into throwing phenotypes, and then tested while in the walking phenotype. Typically, it was found that the visio-motor scale developed while in one phenotype did not transfer to the other: the thrower-turned-walker participants do not show effects of the earlier throwing manipulation on their distance judgments while walkers (Witt et al., 2004), and vice versa (Witt et al., 2010).

In our experiments, however, no behavioral phenotype was turned on, manipulated, switched off, replaced by another, and then examined. Instead, our participants were walker-then-interactor phenotypes throughout. That is, the phenotype we manipulated (the interactor phenotype) was (a) always turned on and (b) always conjoined with the walker phenotype. Thus, by virtue of being conjoined with the interaction system during simulation, the locomotion system was “contaminated” by the constantly-running interaction system. This conjoining led to the effort parameter values instantiated in the interaction system to diffuse into the parameters in the locomotion system. We captured the state of the latter parameters through visual-distance estimates, and we hypothesized that they function, by proxy, as indicators of the amount of effort experienced by the interaction system.

We believe that these subtle design differences render our original results and theoretical arguments immune to Wilson's critiques. Perceived motor effort to interact with in-groups and out-groups can still be a conceptually valid re-characterizations of the cultural construct of interdependence-independence. And, importantly, when viewed in light of the RMC effects, our results can be categorized as belonging to the same class of phenomena explained by Proffitt's theoretical framework. We thank Wilson for providing the opportunity for us to develop this account in greater detail, and we look forward to tests of the proposal.

## Conflict of interest statement

The authors declare that the research was conducted in the absence of any commercial or financial relationships that could be construed as a potential conflict of interest.

## References

[B1] Adam J. J. Nieuwenstein J. H. Huys R. Paas F. G. Kingma H. Willems P. (2000). Control of rapid aimed hand movements: the one- target advantage. J. Exp. Psychol. Hum. Percept. Perform. 26, 295–312 10.1037/0096-1523.26.1.295 10696619

[B2] Ansuini C. Giosa L. Turella L. Altoe G. Castiello U. (2008). An object for an action, the same object for other actions: effects on hand shaping. Exp. Brain Res. 185, 111–119 10.1007/s00221-007-1136-4 17909766

[B3] Cockell D. L. Carnahan H. McFadyen B. J. (1995) A preliminary analysis of the coordination of reaching, grasping, and walking. Percept. Mot. Skills 81, 515–519 10.2466/pms.1995.81.2.515 8570350

[B16] Gentilucci M. Benuzzi F. Gangitano M. Grimaldi S. (2001). Grasp with hand and mouth: a kinematic study on healthy subjects. J. Neurophysiol. 86, 1685–1699 11600632 10.1152/jn.2001.86.4.1685

[B4] Khan M. Mottram T. Adam J. Buckolz E. (2010). Sequential movement with two limbs and the one target advantage. J. Mot. Behav. 42, 325–332 10.1080/00222895.2010.510544 20826424

[B5] Khan M. A. Mourton S. Buckolz E. Franks I. M. (2007). The influence of advance information on the response complexity effect in manual aiming movements. Acta. Psychol. (Amst), 127, 154–162 10.1016/j.actpsy.2007.04.001 17521597

[B6] Lajoie J. M. Franks I. M. (1997). Response programming as a function of accuracy and complexity: evidence lfom latency and kinematic measures. Hum. Mov. Sci. 16, 485–505

[B15] Markus H. Kitayama S. (1991). Culture and the self: implications for cognition, emotion, and motivation. Psychol. Rev. 98, 224–253 10.1037/0033-295X.98.2.224

[B7] Proffitt D. R. Linkenauger S. A. (2013). Perception viewed as a phenotypic expression, in Action Science: Foundations of An Emerging Discipline, eds Prinz W. Beisert M. Herwig A. (Cambridge, MA: MIT Press), 171–197

[B8] Schnall S. Harber K. Stefanucci J. Proffitt D. (2008). Social support and the perception of geographical slant. J. Exp. Soc. Psychol. 44, 1246–1255 10.1016/j.jesp.2008.04.011 22389520 PMC3291107

[B9] Soliman T. Gibson A. Glenberg A. M. (2013) Sensory motor mechanisms unify psychology: the embodiment of culture. Front. Psychol. 4:885 10.3389/fpsyg.2013.00885 24348439 PMC3843157

[B10] Studenka B. E. Seegelke C. Schutz C. Schack T. (2012) Posture based motor planning in a sequential grasping task. J. Appl. Res. Mem. Cogn. 1, 89–95 10.1016/j.jarmac.2012.02.003

[B11] Wilson A. D. (2014). Action scaling of distance perception is task specific does not predict “the embodiment of culture”: a comment on Soliman, Gibson and Glenberg 2013. Front. Psychol. 5:302 10.3389/fpsyg.2014.00302 24795667 PMC4001027

[B12] Witt J. K. Proffitt D. R. Epstein W. (2004). Perceiving distance: a role of effort and intent. Perception 33, 570–590 10.1068/p5090 15250663

[B13] Witt J. K. Proffitt D. R. Epstein W. (2010). When and how are spatial perceptions scaled? J. Exp. Psychol. Hum. Percept. Perform. 36, 1153–1160 10.1037/a0019947 20731519

[B14] van der Wel R. P. Rosenbaum D. A. (2007). Coordination of locomotion and prehension. Exp. Brain Res. 176, 281–287 10.1007/s00221-006-0618-0 16874513

